# Continuous Real-Time Detection of Serotonin Using an Aptamer-Based Electrochemical Biosensor

**DOI:** 10.3390/bios13110983

**Published:** 2023-11-13

**Authors:** Habib M. N. Ahmad, Arturo Andrade, Edward Song

**Affiliations:** 1 Department of Electrical & Computer Engineering, University of New Hampshire, Durham, NH 03824, USA; habib.ahmad@unh.edu; 2 Robert J. & Nancy D. Carney Institute for Brain Science, Brown University, Providence, RI 02912, USA; arturo_andrade@brown.edu; 3 Department of Neuroscience, Brown University, Providence, RI 02912, USA

**Keywords:** serotonin, real-time detection, neurotransmitters, electrochemical sensing

## Abstract

Serotonin (5-HT) is a critical neurotransmitter involved in many neuronal functions, and 5-HT depletion has been linked to several mental diseases. The fast release and clearance of serotonin in the extracellular space, low analyte concentrations, and a multitude of interfering species make the detection of serotonin challenging. This work presents an electrochemical aptamer-based biosensing platform that can monitor 5-HT continuously with high sensitivity and selectivity. Our electrochemical sensor showed a response time of approximately 1 min to a step change in the serotonin concentration in continuous monitoring using a single-frequency EIS (electrochemical impedance spectroscopy) technique. The developed sensing platform was able to detect 5-HT in the range of 25–150 nM in the continuous sample fluid flow with a detection limit (LOD) of 5.6 nM. The electrochemical sensor showed promising selectivity against other species with similar chemical structures and redox potentials, including dopamine (DA), norepinephrine (NE), L-tryptophan (L-TP), 5-hydroxyindoleacetic acid (5-HIAA), and 5-hydroxytryptophan (5-HTP). The proposed sensing platform is able to achieve high selectivity in the nanomolar range continuously in real-time, demonstrating the potential for monitoring serotonin from neurons in organ-on-a-chip or brain-on-a-chip-based platforms.

## 1. Introduction

5-Hydroxytryptamine (5-HT), more commonly known as serotonin, is a signaling biochemical involved in many of the human physiological processes but most notably in the brain function. It is involved in the regulation of many physiological signaling events, including sleep, hunger, and mood control [1]. It plays a crucial role in both the central, as well as the peripheral, nervous system [2,3]. In particular, the role of serotonin in mental and physical health, such as depression [4,5] and cancer [6,7,8,9], has been well documented [10,11]. As a result, there has been a high demand for medical diagnostics based on serotonin detection from biological fluids [12,13,14]. Furthermore, the accurate monitoring of serotonin on a continuous time scale with high temporal resolution can help advance our understanding of the role of serotonin in many neurological disorders.

Various analytical methods, including fluorimetry [15], radio immunoassays [16], enzyme immunoassays [17], chemiluminescence [18], and mass spectrometry [19], have been used for the detection of 5-HT. Although many of these techniques achieve high sensitivity and specificity in detection, they are not ideal for the in situ continuous and real-time measurement of an analyte from neurons or tissue samples due to many factors, including bulkiness of the instrument, long measurement times, and a requirement for sampling and offline analysis, to name a few. Alternatively, an electrochemical sensing approach offers unique advantages in continuous real-time monitoring due to its ease of miniaturization, rapid reaction kinetics, high temporal resolution, and high sensitivity, among others [20,21,22,23,24,25]. For these reasons, the real-time detection of molecules using electrochemical sensing platforms has been largely successful [26,27].

Since 5-HT is an electroactive compound, the direct oxidation of 5-HT at the electrode surface can be performed to electrochemically quantify its concentration [26,27]. However, one critical challenge with the electrochemical detection of serotonin is the possible interference in the voltammetry signals due to the presence of other electroactive molecules with similar redox potentials. For example, both ascorbic acid (AA) and dopamine (DA) have oxidation potentials that closely overlap with that of 5-HT on a conventional gold (Au) or a glassy carbon electrode (GCE) [28,29]. DA, 5-HT, and AA have similar oxidation potentials at most solid electrodes, and therefore, the selective quantification of these species is a great challenge due to their overlapping signals [24,30]. DA shows a sluggish and much smaller cyclic voltammetry (CV) peak response with a ΔEp of 0.35 V at a bare GCE vs. Ag/AgCl in the phosphate buffer (PBS) solution (pH 7.0) [30]. The voltammetric peak of 5-HT in neutral, pH 7.0 PBS appears at about 0.46 V at the bare GCE, and the peak appears to be broad, indicating a slow electron-transfer kinetics [30]. On a bare GCE, AA shows a broad and irreversible oxidation peak at 0.35 V vs. Ag/AgCl in a neutral pH PBS buffer [30]. L-tryptophan (L-TP) and 5-hydroxytryptophan (5-HTP), which are precursors of 5-HT and 5-hydroxytryptamine (5-HTA), a metabolite of 5-HT, are also electroactive molecules [31,32,33,34]. Therefore, there is a critical need to develop an electrochemical biosensing platform that can monitor 5-HT on a continuous time scale while ensuring target selectivity and sensitivity.

Aptamer-based electrochemical sensing has been and continues to be a promising technology in monitoring a variety of biomarkers, including biochemicals, drugs, proteins, and pathogens. In particular, the aptamer’s ability to measure the analyte continuously without a need for sensor regeneration allows for aptamer-based sensors to be used in real-time sensing applications [35,36]. Furthermore, aptamers promote target selectivity by preferentially binding with the target analyte. This is especially useful when trying to detect non-electroactive targets using electrochemistry. However, even in the case where the analyte is electroactive (such as serotonin), and therefore, direct oxidative detection is achievable, the incorporation of aptamers into the sensor electrode can still be beneficial because it can potentially suppress interfering signals from other species with similar redox potentials. Previously, aptamers have been used as receptor molecules in electrochemical sensors for serotonin (5-HT) detection [37]. The SELEX (systematic evolution of ligands by exponential enrichment) technique was employed to select such receptors from a pool of single-stranded nucleic acid oligomers. In particular, Nakatsuka and others have previously produced the initial aptamer developed exclusively for serotonin (5-HT) detection, presenting a strong affinity in a fluorescence assay with a dissociation constant (K_d_) of 30 nM [38]. Employing field-effect transistors and nanopipette devices, this aptamer has also been successfully used in the study of serotonin and other neurotransmitters [38,39,40].

**Figure 1:**
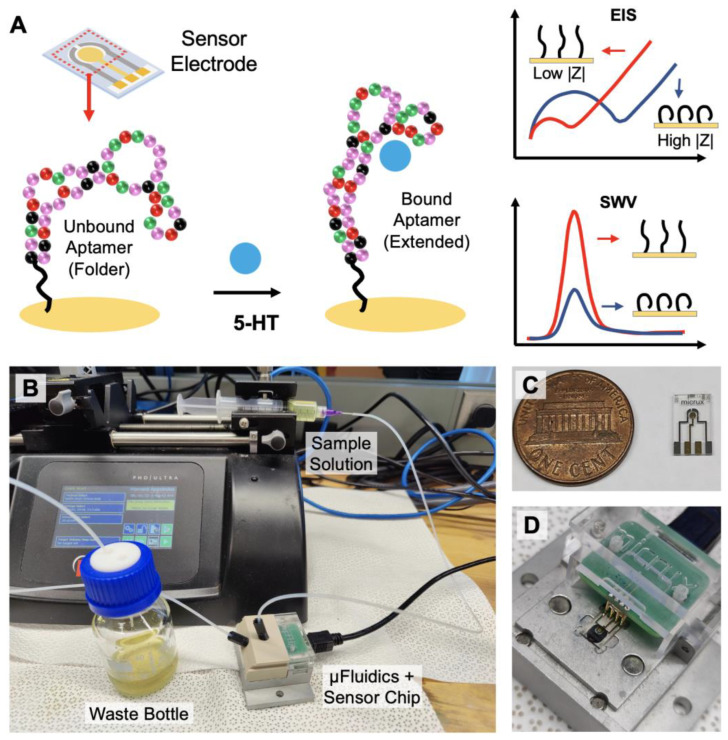
Overview of the aptamer-based electrochemical serotonin sensing: (**A**) serotonin-binding aptamers immobilized on the electrode undergo conformational change from folded to extended upon target recognition. EIS and SWV techniques can be applied for serotonin quantification; (**B**) for continuous real-time monitoring, the sample buffer solution is injected with a syringe pump into the microfluidic device containing the sensor chip; (**C**) image of the 3-electrode sensor chip; (**D**) the electrodes on the chip are securely connected to the terminals of the potentiostat via spring-loaded pins.

In this paper, we demonstrate an electrochemical sensing strategy that can monitor serotonin continuously in real-time with high sensitivity and selectivity (Figure 1). Aptamers that have specific affinity to 5-HT have been immobilized on the surface of the electrode to enhance target selectivity and specificity while minimally compromising the temporal resolution. Utilizing the aptamer’s ability to change its conformation upon specific target binding, electrochemical impendence spectroscopy (EIS) and square wave voltammetry (SWV) can be applied as sensing techniques for detecting serotonin under static environments (Figure 1A). Furthermore, a fixed-frequency EIS is used for monitoring dynamic changes in the serotonin concentration in real-time [41]. Using a continuous flow microfluidic setup, varying concentrations of 5-HT can be introduced into the system for the time-dependent detection of 5-HT (Figure 1B–D). Based on the results presented in this work, we demonstrate that the proposed sensing strategy has the potential to monitor the changing dynamics of serotonin continuously in real-time from physiological samples.

## 2. Materials and Methods

### 2.1. Chemical Reagents

The high-performance liquid chromatography (HPLC)-purified 44-mer 5-HT-binding DNA aptamers, the IDTE (10 mM Tris, 0.1 mM EDTA) resuspension buffers (pH 8), the folding buffers, and the reducing buffers were purchased from Base Pair Biotechnologies, Inc. (Pearland, TX, USA). The aptamer’s affinity has been thoroughly characterized by the manufacturer using Microscale Thermophoresis (MST) Analysis and is shown to be specific toward serotonin (5-HT). The sequence of the aptamer is as follows [38]:

5′-ThiolC6-HO-C6-S-S-C6-CGA CTG GTA GGC AGA TAG GGG AAG CTG ATT CGA TGC GTG GGT CG-3′.

The molecular weight and length of the aptamers are 14,102.3 g/mol and 44 nucleotides, respectively. The aptamers were synthesized with a thiol functional group termination at the 5′ end. The thiol group can form a uniform and compact self-assembled monolayer (SAM) of the aptamers on an Au electrode.

The following chemicals (ACS grade) were obtained from Sigma-Aldrich Inc. (St. Louis, MO, USA) and used as received: potassium hexacyanoferrate (II) trihydrate, potassium hexacyanoferrate (III), norepinephrine bitartrate salt, 5-hydroxy-L-tryptophan, L-tryptophan, 5-hydroxyindole-3-acetic acid, magnesium chloride, Tris(2-carboxyethyl) phosphine hydrochloride (TCEP). Serotonin hydrochloride, 98%, and dopamine hydrochloride, 99%, were purchased from Alfa Aesar (Tewksbury, MA, USA); 10X PBS Buffer (pH 7.4) was purchased from Thermo Fisher Scientific (Waltham, MA, USA). 6-Mercapto-1-hexanol was purchased from TCI America (Portland, OR, USA).

The amine coupling kit for small molecule immobilization and the high sensitivity carboxyl sensors for the surface plasmon resonance studies were purchased from Nicoya Lifesciences (Kitchener, ON, Canada).

### 2.2. Experimental Apparatus

All electrochemical measurements were obtained using the Biologic VSP potentiostat (Seyssinet-Pariset, France). The electrochemical sensor electrodes, the microfluidic chamber, and interfaces were purchased from Micrux Technologies (Gijón, Spain). The localized surface plasmon resonance (LSPR) experiments were performed with a Nicoya OpenSPR benchtop instrument. The syringe pump systems for the microfluidic experiments were purchased from Harvard Apparatus (Holliston, MA, USA). The XPS (X-ray Photoelectron Spectroscopy) characterization was performed using the Kratos Analytical (Manchester, UK) Axis Supra XPS system.

### 2.3. Methods

#### 2.3.1. Protocol for XPS Study

A glass chip with a Ti/Au layer of 50 nm/150 nm was used for the XPS studies. Prior to XPS analysis, a self-assembled monolayer of the thiolated aptamer was first formed on the Au surface. The XPS spectra were analyzed for the aptamer-attached gold surface before and after exposure to 5-HT. The AXIS Supra instrument was configured with a dual Al Kα/Ag Lα (hν = 2984.2 eV) monochromatic X-ray source. These high-energy photons have the capacity to ignite photoelectrons from higher binding energy core-levels that are otherwise difficult to excite. Furthermore, as the kinetic energy of the core level electrons grows, so does the informational depth, allowing for larger sampling depth in comparison to standard Al Kα. During the analysis, the residual pressure in the analysis chamber was less than 1 × 10^−9^ Torr. At least three survey spectra were obtained for each specimen and utilized to study the surface chemical composition. A magnetic charge compensation method was used to adjust for the surface charge. The acquisitions had a 90° take-off angle with respect to the sample surface. To minimize X-ray degradation, the collection period was kept under 20 min per sample, and wide and core region spectra were recorded on distinct sample sites. The data were recorded, and the XPS peaks were analyzed with ESCApe Acquisition and Processing software (Kratos Analytical, Manchester, UK). The atomic percentages (at%) were computed from the experimentally measured peak intensities and normalized using Kratos Analytica’s atomic sensitivity parameters. Peak fitting was carried out without any preparatory filtering. After a Shirley-type background subtraction, symmetric Gaussian–Lorentzian product functions (70% Gaussian and 30% Lorentzian) were utilized to approximate the line forms of the fitted components.

#### 2.3.2. Protocol for SPR Study

High sensitivity carboxyl sensor chips were used for the SPR experiment. An air-dried sensor chip was loaded into the instrument, and optical references were set according to the manufacturer’s manual. Initially, the sensor surface was cleaned with 10 mM HCl (pH 2) at a flow rate of 150 µL/min. Then, the carboxyl groups on the chip surface were activated with a 1-ethyl-3-(3-dimethylaminopropyl)-carbodiimide (EDC) and *N*-hydroxysuccinimide (NHS) mixture to chemically couple the ligand via its primary amine groups. The EDC-NHS mixture was flown at a rate of 20 µL/min and allowed to interact with the surface for at least 5 min. The reaction that occurs when the EDC/NHS combination is introduced onto the carboxyl sensor surface produces succinimide esters. EDC and NHS are highly specific for the carboxyl surface to form an NHS ester that allows for primary amine conjugation at a physiological pH [42]. Serotonin (5-HT) hydrochloride was diluted in the immobilization buffer at a 1 µM concentration and injected at a 20 µL/min flow rate. The -NH_2_ groups from 5-HT were coupled with -COOH groups of the activated sensor surface, which resulted in successful immobilization (see Supplementary Information Figure S7). To prevent further coupling and to reduce non-specific binding, the remaining active carboxyl groups were deactivated with a blocking solution. For target affinity characterizations, various concentrations of 5-HT aptamer solutions were used for the association phase, 1X PBS buffer with 2 mM MgCl_2_ (running buffer) was used for the dissociation phase, and 10 mM NaOH was used for the sensor regeneration phase.

#### 2.3.3. Electrochemical Sensor Preparation

Microscale 3-electrode sensor chips (ED-SE1-AuPt, Micrux technologies) containing a gold working electrode (WE) and platinum reference (RE) auxiliary (AE) electrodes were electrochemically pre-cleaned with 0.05 M H_2_SO_4_ by repeatedly running cyclic voltammetry between −1.0 V and +1.3 V with a scan rate of 0.1 V/s. Afterward, the electrodes were rinsed with isopropyl alcohol and deionized water, respectively. Once fully dried, the electrodes were functionalized with aptamers before sensing experiments were conducted.

#### 2.3.4. Aptamer Suspension on the Electrochemical Sensors

The aptamer stock solutions were prepared by mixing 35.4 nmol of thiolated aptamers with 354 μL of the resuspension buffer from the aptamer manufacturer, which resulted in a final concentration of 100 µM for the aptamer stock solution. The disulfide bond must be reduced before deposition since the manufacturer provides the aptamers in the oxidized form, which does not efficiently immobilize onto the gold surface. The resuspended aptamers were reduced with TCEP maintaining the ratio of 50 μM of aptamer to 10 mM TCEP. The reduced aptamers were further diluted to the working concentration by mixing them with 1X PBS buffer with 2 mM MgCl_2_. The MgCl_2_ is added to prevent non-specific electrostatic interactions. Higher amounts of MgCl_2_ may be detected in tests identifying biomarkers on the surface of malignant cells because metal cations can aid in disguising the negative charge of the DNA backbone [43,44]. This may also explain why variations in the magnesium chloride concentration affect binding affinity [45,46,47]. Diluted aptamers were heated at 95 °C for 5 min and cooled down to room temperature before attaching them to the Au surface. After rinsing the electrochemically active Au electrodes with deionized water, 10 μL of 1 μM thiolated DNA aptamer solution was drop-casted onto the Au electrode, which was kept in the dark overnight (~18 h). The sensor chip was then washed with ultra-pure water and back-filled with 1 mM 6-mercapto-1-hexanol (MCH) for 30 min and again washed with ultra-pure water before proceeding with the electrochemical experiment.

#### 2.3.5. Electrochemical Detection for the Static Measurements

The electrolyte used in the electrochemical experiment consisted of 5 mM K_4/3_Fe(CN)_6_ and 2 mM MgCl_2_ dissolved in 1X PBS with pH 7.1. The electrolyte was prepared fresh for each experiment and purged with N_2_ to remove dissolved oxygen, though N_2_ purging did not affect the results (Figure S11 in Supplementary Material) significantly. Two electrochemical measurement techniques, EIS and SWV, were used for the characterization of the surface-immobilized aptamers, as well as for the 5-HT detection. For EIS, the following parameters were used: working electrode potential, E_we_ = 0.025 V vs. reference, scanning frequency range f_i_ = 100 KHz, f_f_ = 1 Hz, sinusoidal AC voltage amplitude V_a_ = 10 mV. For SWV, the following parameters were used: scanning voltage range was from E_i_ = −0.3 V vs. the reference to E_V_ = 0.7 V vs. the reference, pulse height P_H_ = 25 mV, pulse width P_W_ = 0.6 ms, step height, S_H_ = 5 mV. Experimental parameters are discussed further in detail in the Supplementary section.

#### 2.3.6. Continuous Real-Time Monitoring for Dynamic Measurements

A single-frequency EIS method was used for the continuous and real-time detection of 5-HT under a microfluidic environment. A frequency of 10 Hz was chosen for maximum sensitivity. All microfluidic measurements were performed with a fluid flow rate of 20 μL min^−1^. Different concentrations of the analyte were introduced every 400 s. The raw measurement curves were smoothened with the Savitzky–Golay method with a polynomial order of 2. After each analyte injection, the EIS measurements were averaged for all data points between the 150th and 250th seconds to obtain the average sensor response for the analyte detection.

## 3. Results

### 3.1. Sensor Electrode Surface Chemistry Characterization

#### 3.1.1. Confirmation of Self-Assembled Monolayer on the Electrode

**Figure 2:**
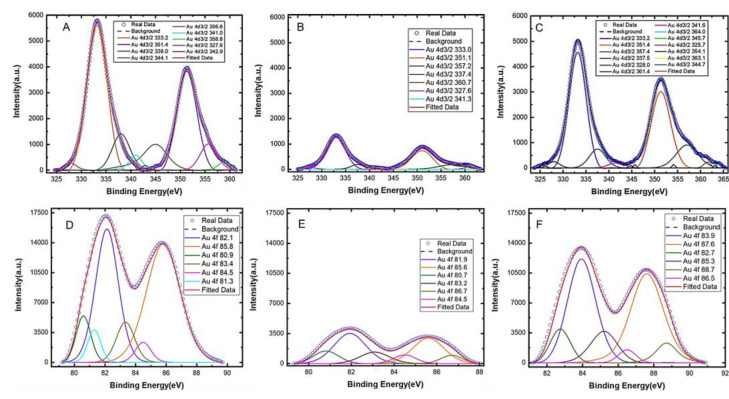
XPS spectra of the Au4d_3/2_ peaks for (**A**) the bare gold electrode, (**B**) the electrode after aptamer immobilization, and (**C**) the aptamer-modified electrode after serotonin (5-HT) exposure. The XPS spectra for the Au4f peaks are also shown for (**D**) the bare electrode, (**E**) after aptamer immobilization, and (**F**) after serotonin 5-HT exposure to the modified electrode.

To verify and characterize the immobilization of aptamers on gold electrodes, X-ray photoelectron spectroscopy (XPS) analysis was performed on the modified electrode to characterize its surface chemistry. After aptamer functionalization, a decrease in the gold content (Figure 2) was observed due to the self-assembled monolayer of aptamers covering the electrode surface. Furthermore, a significant increase in the peak related to the C-O bonds (286.8 eV) in the C1s core level (Figure S1) was observed, which is attributed to the formation of thiol bonds [48]. These findings point to the presence of aptamers on the gold surface covalently anchored via thiol chemistry [49]. Also, increases in the oxygen (Figure S2), sulfur (Figure S5), and phosphorus (Figure S6) content were observed for the modified electrode due to the presence of those elements in the DNAs. The increase in these elements further confirm the presence of a significant number of aptamers anchored onto the electrode [38,50,51]. A detailed XPS analysis of the electrode surface is provided in the Supplementary Material.

#### 3.1.2. Thiol Chemisorption on the Gold Surface

The analyses of the C1s and S2p core-level spectra yielded additional information on the thiol bonds. Because of the existence of the element at 161.9 eV in the high-resolution XPS spectrum of S2p (Figure S1B), it can be concluded that the functionalization occurred via S-Au chemisorption. In fact, the S2p peak can be fitted with two components, one for each spin-orbit splitting doublet S2p_1/2_ and S2p_3/2_. The first component is associated with bound sulfur and is centered at about 162 eV; the second (beyond 164 eV) is associated with the presence of some unbound sulfur on the surface [52]. While the first component is related to the thiol chemisorption, the second highlights the presence of weakly bound (physiosorbed) thiols on the surface even after thorough rinsing with ethanol and water. This component may be due to the thiolated aptamer molecules that form the self-assembled monolayer on the surface [53,54].

#### 3.1.3. Assessment of Aptamer Conformation Change upon Serotonin Binding

The XPS analysis also provides further insight into the surface morphology of the electrode before and after target binding. The peak intensities of the spectra for Au4d_3/2_ and Au4f (Figure 2) increased significantly (panels C and F) after serotonin was allowed to bind with the aptamers on the gold surface compared to the spectra before serotonin binding (panels B and E). An increased spectral intensity after target binding suggests that the conformations of the aptamers have changed upon serotonin exposure. It is worth noting that, since XPS analysis is performed in a vacuum, the conformation of the aptamers (whether folded or extended) in solution cannot be determined using the XPS spectra. Therefore, the increased spectral intensity after serotonin exposure should not be interpreted as an aptamer conformation change from a folded to extended state, but instead, it is merely an indication that some form of conformation changes has occurred upon target exposure. Furthermore, no MCH backfill was applied on the gold surface and therefore, any non-specific adsorption that might have occurred would have further reduced the intensity of the spectra (rather than increasing them) because it would have further passivated the gold surface preventing the XPS from detecting gold. Based on our analysis, we conclude that the change in the spectral intensity after serotonin exposure is primarily due to the binding-induced reorientation of the aptamers immobilized on the gold surface.

### 3.2. Characterization of the Aptamers Using Surface Plasmon Resonance

**Figure 3:**
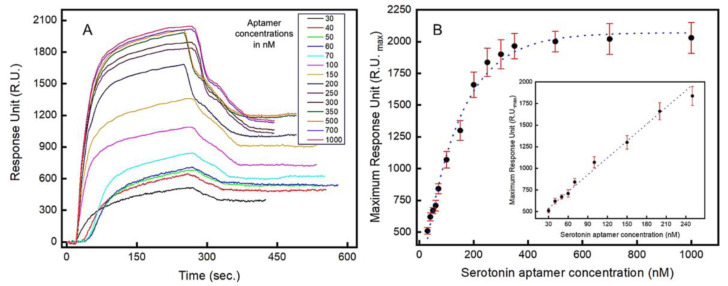
Localized surface plasmon resonance (LSPR) study to characterize the aptamers’ binding kinetics to serotonin (5-HT): (**A**) real-time LSPR responses to varying concentrations (in nM) of serotonin (5-HT) showing association and dissociation between the aptamers and serotonin (5-HT); (**B**) Langmuir isotherm showing the LSPR response vs. serotonin (5-HT) concentration. Inset: LSPR response in the linear range (30–250 nM). Error bars indicate 1 standard deviation (n = 3).

Localized surface plasmon resonance (LSPR) is a highly sensitive optical method for the real-time analysis of molecular interactions and, in this work, LSPR is used to characterize the binding kinetics of the aptamers. The real-time LSPR responses for the serotonin–aptamer interaction on the gold surface is shown in Figure 3A. The serotonin molecules were immobilized on the surface of the LSPR chip, and varying concentrations of aptamer-containing solutions (30–1000 nM) were introduced. Both association and dissociation phases are presented where the rate of association is significantly more rapid than the dissociation rate (Figure 3A). The maximum optical intensity around 2030 RU was estimated when 1000 nM of aptamer solution was injected into the flow cell (Figure 3B). A linear relationship (R^2^ = 0.985) between the LSPR signal intensity and the aptamer concentrations introduced was observed in the range between 30 nM and 250 nM (Figure 3B inset). From the LSPR analysis, a dissociation constant (K_d_) of 87 nM was obtained, which corresponds to the concentration at which 50% of the maximum LSPR intensity is observed. The limit of quantification (LOQ) and the limit of detection (LOD) were estimated to be 51.39 nM and 16.95 nM, respectively. Figure S8 shows the real-time concentration-dependent response of the serotonin–aptamer interaction.

### 3.3. Aptamer Surface Coverage Estimation

The density of aptamers immobilized on the electrode surface can significantly influence the sensing performances, including the sensitivity, detection limit, and response time among others. If aptamers are overcrowded on the electrode, the binding constants for the target molecule can be negatively affected due to the high intermolecular interactions between neighboring aptamers [55]. Conversely, sparsely distributed aptamers will result in a poor signal-to-noise ratio and sensitivity to the target. Therefore, to achieve an optimal aptamer surface density, we performed electrochemical analyses based on electrodes with varying degrees of surface coverage by the aptamers. Several different concentrations of thiol-terminated aptamer solutions, ranging from 0.1 to 20 µM, were used to immobilize the aptamers on the gold electrodes. A fixed incubation time (18 h) was used for all electrodes to minimize time-dependency of the aptamer functionalization process. To ensure proper secondary structures are formed in the aptamers, the heating and cooling steps were applied prior to immobilization. The backfilling of MCH covers the open regions of the electrode not occupied by the aptamers, which reduces non-specific adsorption and therefore noise in the electrochemical measurements.

**Figure 4:**
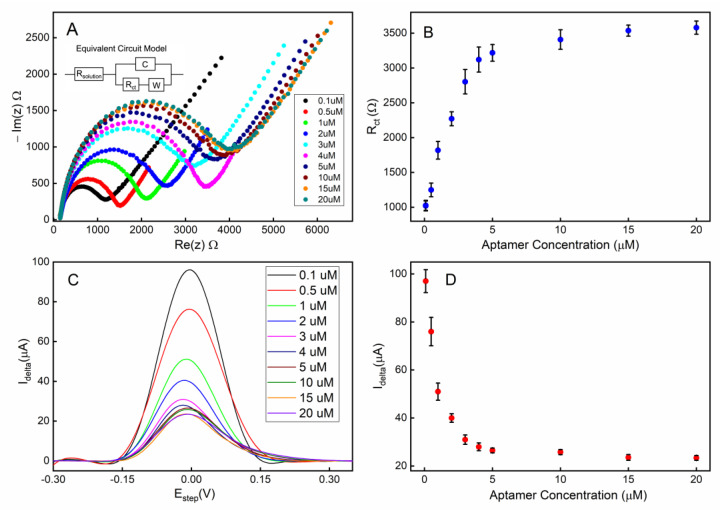
Electrode surface coverage characterization via electrochemical impedance spectroscopy (EIS) and square-wave voltammetry (SWV): (**A**) the Nyquist plot for the electrode when different concentrations of aptamers were used during the immobilization step; (**B**) the calibration curve corresponding to the Nyquist plot; (**C**) the SWV plot of the electrode when different concentrations of aptamers were used; (**D**) the calibration curve corresponding to the SWV plot. All error bars indicate 1 standard deviation (n = 3).

The electrochemical impedimetric behavior of the aptamer-attached electrode using [Fe(CN)_6_]^4-/3-^ as a redox couple with 2 mM MgCl_2_ is shown in Figure 4A. The immobilization of the aptamers resulted in an increased impedance due to the incorporation of aptamers that hinder the charge transfer at the solution-electrode interface. Moreover, the single-stranded DNA-based aptamers carry negatively charged phosphate backbones, which further increase the potential energy barrier that the free electrons must overcome to achieve Faradaic current flow. Figure 4B shows that the charge transfer resistance (R_ct_) linearly increased for the aptamer concentration range of 0.1–4 µM suggesting linearly increasing surface coverage by the aptamers within that concentration range. As the aptamer concentration was further increased beyond 4 µM, the R_ct_ was saturated indicating overcrowding of the aptamers and possibly reaching the maximum occupancy of aptamers on the electrode.

Square wave voltammetry (SWV) was employed as a complementary validation technique along with EIS for electrode surface characterization. The SWV has the capacity to minimize the capacitive current, as well as parasitic currents, due to dissolved oxygen reduction. SWV measures currents from both positive and negative potential pulses sequentially, and the registered current is the subtraction of the oxidation and reduction currents. This leads to higher current density in SWV compared to that in CV [56]. A clear correlation can be observed between the aptamer surface coverage density (described by the concentration of the aptamer solution used during functionalization) and the redox current peak (Figure 4C,D). These data are also in agreement with the concentration-dependent surface coverage profile obtained with EIS in Figure 4B.

To achieve the optimum surface coverage of aptamers on the electrode surface, we chose an immobilization condition that would yield approximately 50% of the maximum charge transfer resistance (R_ct_) or peak redox current (I_delta_) from EIS (Figure 4B) or SWV (Figure 4D) measurements, respectively. Based on our observation, using a 1 µM aptamer concentration with a deposition time of 18 h would achieve an optimum surface coverage of aptamers for the electrochemical detection of serotonin.

### 3.4. Detection of Serotonin under a Static Environment

**Figure 5:**
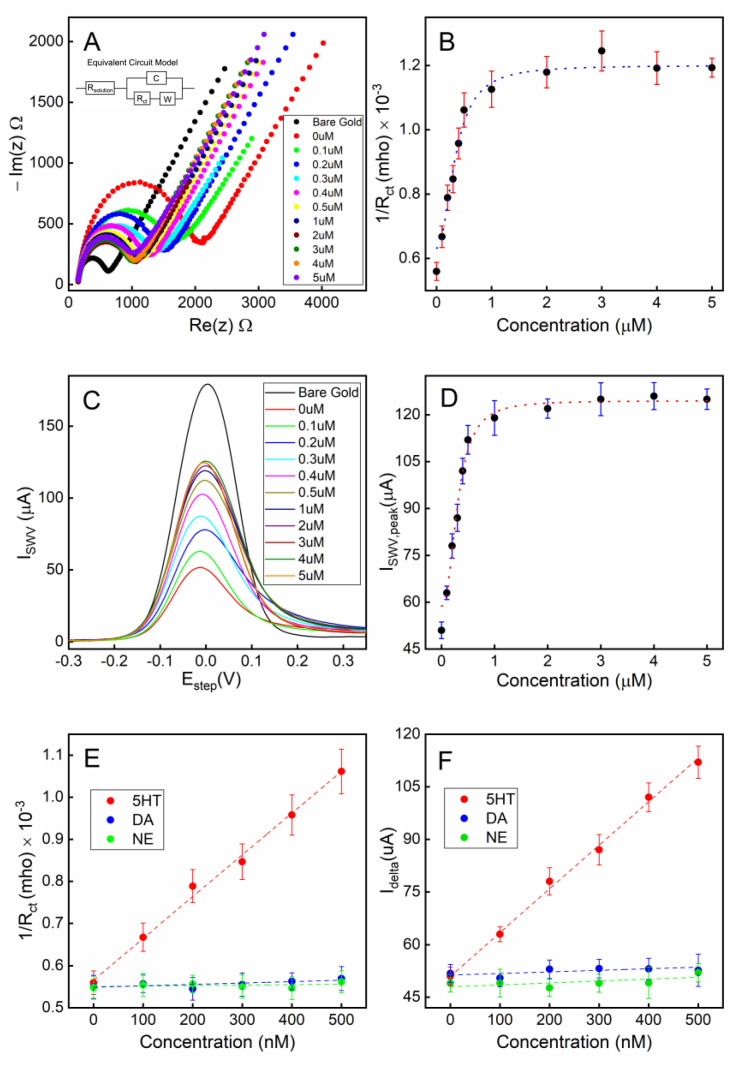
Detection of serotonin (5-HT) under a static fluidic environment: (**A**) Nyquist plots of EIS under various concentrations of serotonin (5-HT) exposed to the sensor; (**B**) a calibration curve showing the conductance vs. serotonin (5-HT) concentration; (**C**) SWV responses of the sensor to various concentrations of serotonin (5-HT); (**D**) a calibration curve showing the peak current of the SWV curve vs. serotonin (5-HT) concentration; (**E**) a calibration curve for EIS-based sensing in the linear range; (**F**) a calibration curve for SWV-based sensing in the linear range. All error bars indicate 1 standard deviation (n = 3).

To characterize the serotonin detection capability of our electrochemical sensor, static measurements (stationary fluid) were first performed. The aptamer-attached gold electrode, with the unoccupied gold surface backfilled with MCH, was first exposed to various concentrations of serotonin (0.1–5 µM) in 1X PBS for 30 min to allow target-receptor binding. After gently rinsing the electrode, the electrochemical measurements were performed on the sensor under the sensing electrolyte. As shown in the Nyquist plot in Figure 5A for the EIS measurements, the charge transfer resistance (R_ct_) of the Randles circuit fitting model is strongly correlated to the concentration of serotonin for concentrations in the range 0.1–5 µM (Figure 5B). This plot suggests that as the conformations of the aptamers change from a collapsed to extended shape resulting from the target-specific binding, the impedance at the solution–electrode interface is decreasing due to the unfolding of the aptamers. As the exposed serotonin concentration continues to increase, the Rct reaches a plateau as most aptamers have bound to the 5-HT molecules, and no further conformational changes occur at the electrode surface.

A square wave voltammetry (SWV) technique was also applied to the electrode to characterize the voltammetry response of the sensing electrode. A clear correlation between the peak current of the SWV and the concentration of 5-HT can be seen in Figure 5C,D.

The limit of quantification (LOQ) and the limit of detection (LOD) were calculated using the formulae $\frac{10\sigma }{S}\;and\;\frac{3.3\sigma }{S}$, respectively where σ is the standard deviation of the response and S is the slope of the calibration curve. LOQs were found to be 0.135 µM and 0.117 µM, while LODs were calculated as 41 nM and 32 nM for the calibration curves of EIS and SWV, respectively.

The calibration curves shown in Figure 5B,D were both fitted with nonlinear least-squares fitting to the Hill equation (Equation (1)), with the Hill coefficients (*n*) set as a variable [57,58].

(1) $$I=I_{i}+\left(I_{f}-I_{i}\right)\frac{C^{n}}{{{(k}_{d,eff})}^{n}+C^{n}}$$

where *I* is the data point on the calibration curve measured as a function of target concentration *C*; *I_i_* and *I_f_* are the minimum and maximum measured data, respectively; and *k_d,eff_* represents the effective dissociation constant of the binding receptor. Based on the curve-fitting of our measurements, *k_d,eff_* was found to be 343.54 ± 61.03 nM and 306.76 ± 41.54 nM according the results from our impedimetric and voltametric techniques, respectively.

The aptamers exhibited excellent target selectivity against the potential interfering species dopamine (DA) and norepinephrine (NE) when tested under a static fluidic environment (Figure 5E,F).

### 3.5. Continuous Detection of Serotonin Using a Microfluidic System

In this section, we investigate our sensor’s capability of performing the continuous and potentially real-time monitoring of serotonin from an environment with dynamically changing serotonin concentrations. To achieve optimal temporal resolution and the sampling time of the sensor, we employed single frequency electrochemical impedance spectroscopy (SF-EIS) as a method of analyte detection. Unlike EIS, which requires a frequency sweep, the fixed frequency nature of SF-EIS technique allows for the sensing to be performed at a high sampling rate. The optimal frequency at which to operate SF-EIS for our sensor was chosen to be 10 Hz based on the Bode plot (Figure S9) and a selection of the frequency that showed the highest sensitivity in the sensor responses. A microfluidic system housing the 5-HT sensor chip was employed to enable a real-time concentration change in 5-HT (Figure S10). To simulate a rapid concentration change, the syringes containing various concentrations of 5-HT were swapped in real-time, which caused a step change in the 5-HT concentration being introduced into the microfluidic channel by the syringe pump.

**Figure 6:**
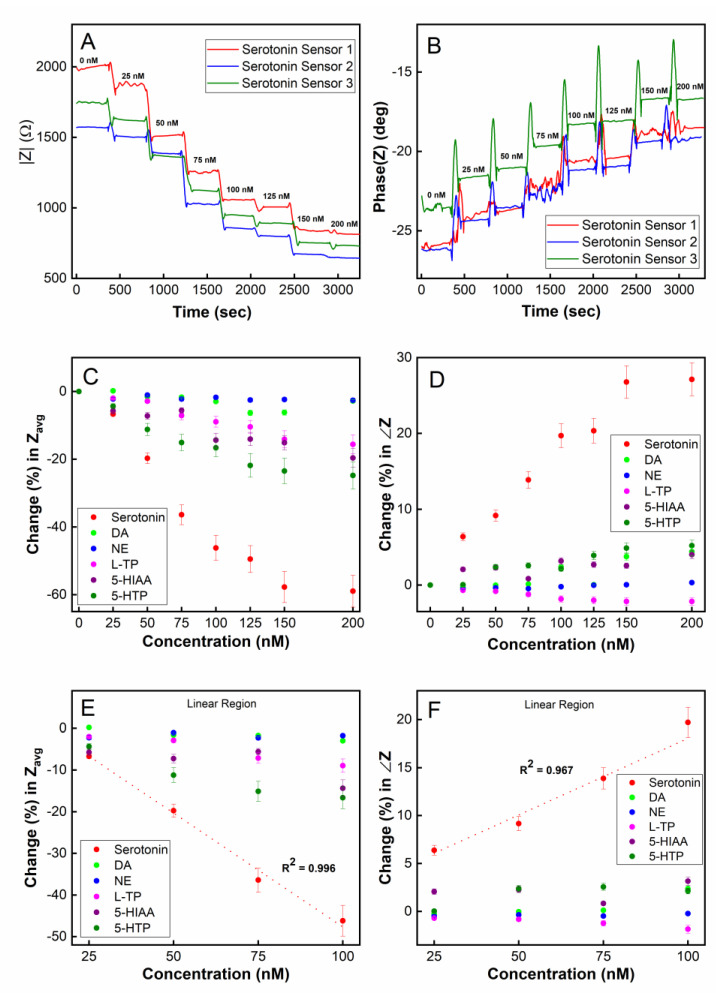
Real-time detection of serotonin (5-HT) using single-frequency EIS (SF-EIS): (**A**) plot of impedance magnitude (|Z|) vs. time; (**B**) plot of impedance phase (∠Z) vs. time; (**C**) target selectivity test in a real-time sensing mode using averaged |Z| (Z_avg_) as a sensor readout; (**D**) target selectivity test in real-time using ∠Z as a sensor readout; a linear detection range of 25–100 nM was observed using (**E**) the impedance magnitude (|Z|) and (**F**) the impedance phase (∠Z).

As shown in Figure 6A, using the magnitude of impedance |Z| as a readout, the sensor was able to monitor real-time changes in the 5-HT concentration in the range of 0–150 nM. When a step change in concentration occurs, the |Z| measurement immediately responds and reaches a steady-state value within approximately 1 min, which can be interpreted as the sensor’s temporal resolution. This time resolution is in agreement with our real-time SPR measurements (Figure 3) where the steady-state for the association between the aptamers and 5-HT was reached approximately 80–100 s after target introduction to the SPR chip. The stabilization time for dissociation was approximately 60–80 s based on our SPR data. Given such binding kinetics of the aptamers, it is reasonable to expect that the highest achievable temporal resolution for 5-HT sensing would be in the neighborhood of 1 min. It should be noted that the binding kinetics observed in SPR may be different from that in the electrochemical sensor since, in the SPR system, 5-HT is immobilized on the sensor chip’s surface while in the electrochemical sensor, the aptamer is immobilized on the electrode. Although the immobilization of 5-HT on the SPR chip’s surface may interfere with the aptamer’s affinity for 5-HT (due to its amine group binding to the chip via EDC/NHS chemistry), our SPR result indicates that there is still a strong affinity between 5-HT and the aptamer even for immobilized 5-HT molecules. The phase of the impedance for the SF-EIS under step changes in the 5-HT concentration was also plotted (Figure 6B) showing concentration-dependent responses. However, compared to the |Z| plot, a substantially higher noise was recorded during the transient period due to a step change in the concentration.

To test the chemical selectivity of our sensor using the microfluidic platform, the sensor was exposed to five potential interfering species of serotonin, namely, dopamine (DA), norepinephrine (NE), L-tryptophan (L-TP), 5-hydroxyindoleacetic acid (5-HIAA), and 5-hydroxytryptophan (5-HTP). Figure 6C (Z magnitude plot) and Figure 6D (Z phase plot) show that the sensor was highly selective toward serotonin and was minimally influenced by other chemical species when measured continuously in real-time. A linear detection range from 25 nM to 100 nM was observed, demonstrating the capability for double-digit nanomolar detection in a real-time environment (Figure 6E,F). The limit of detection (LOD) was calculated to be 5.6 nM in a real-time continuously flowing microfluidic environment employing the SF-EIS technique.

## 4. Discussion

Serotonin levels were found in platelet-poor plasma to be in the 1–100 nM range where a healthy person’s whole blood can contain serotonin levels in the 0.5–1.7 µM range [59,60]. Researchers have found that patients suffering major depression typically have lower serotonin levels, of around 0.3 µM, in their blood compared to a healthy individual level of 1.15 µM [61]. Interestingly, Type 2 diabetic patients with chronic kidney diseases also show lower levels of serotonin, of around 0.5 µM, in their blood sample [62]. Serotonin levels were found to be about 280 µM over the duration of 24 h in urine samples of patients with carcinoid tumors [63]. Normal serotonin levels have been found to be around 300–1650 nM in human urine samples and less than 0.0568 nM in cerebrospinal fluid (CSF) [60,64,65]. Therefore, the linear concentration range detectable with our proposed aptamer-based sensor is physiologically relevant to clinical applications where abnormal serotonin levels can be measured electrochemically. With respect to other available serotonin sensors (Table 1), our sensor demonstrated competitive analytical performances when compared against the majority of recently published work. In terms of the continuous real-time monitoring of serotonin, our sensor’s time resolution of approximately 1 min is comparable to other real-time serotonin sensors in the literature, which were in the range of 30 s^−1^ min. Moreover, our sensing platform was found to be highly sensitive and specific to 5-HT. Some cross-reactivity exists for other potentially interfering molecules, such as 5-HTP, 5-HIAA, and L-TP, due to the similarities in chemical structures, suggesting that the aptamers exhibit a certain degree of affinity toward these species. Therefore, further optimization in the aptamer design may be necessary to further improve target selectivity. Nevertheless, the aptamer’s response to 5-HT is overwhelmingly high compared to other non-specific chemicals. Given our sensor’s excellent performance in the PBS environment, our future work includes the validation of our serotonin biosensor in a real physiological sample, such as CSF in a dynamically flowing environment, and comprehensive selectivity testing with mixed interfering species.

## 5. Conclusions

A novel DNA aptamer-based label-free electrochemical sensor was successfully developed and tested in real-time to detect serotonin (5-HT). Both impedimetric and voltametric methods were used to characterize the aptamer-modified surface density and the sensing of 5-HT. The aptamer surface density is crucial for ensuring sensor sensitivity. The developed platform offers a dual-mode electrochemical method (EIS and SWV) to optimize the surface density for the maximum electron transfer rate at the electrode–electrolyte interface. The dual-mode platform was used for estimating the dissociation constant for the proposed sensor, and values were found to be 343.54 ± 61.03 nM and 306.76 ± 41.54 nM for EIS and SWV, respectively. Single-frequency EIS (SF-EIS) was successfully implemented for the real-time 5-HT detection with reasonable temporal resolution on a microfluidic system. The sensor’s selectivity was characterized against DA, NE, L-TP, 5-HIAA, and 5-HTP, and the sensor was highly selective toward 5-HT, indicating the specificity of the aptamer’s binding kinetics. Given the sensor’s sensitivity, selectivity, range of detection, and temporal resolution, our sensing platform has the potential to be used for studying the serotonin dynamics in animal models or in a clinical setting.

## Figures and Tables

**Table 1:** Comparison with other recently published serotonin sensors.

Sensing Platform	Linear Range	LOD	Reference
Aptamer/AuNPs	750 nM–2.5 μM	300 nM	[66]
Aptamer/SWCNT	100 nM–1 μM	-	[67]
Aptamer/Gold	1–100 μM	300 nM	[68]
GR/p-AHNSA/SPCs	0.05–150 µM	3 nM	[69]
AgNP/L-Cys/MXene	500 nM–150 μM	80 nM	[30]
ssDNA-SWCNT	0.1–50 μM	-	[70]
GR-FeTSPc	0.1–100 μM	20 nM	[71]
Aptamer/Nanopipette	1–100 pM	1 pM	[39]
Aptamer/MCH	25–100 nM	5.6 nM	This Work

## Data Availability

The data presented in this study are available in this article.

## Supplementary Materials

The following supporting information can be downloaded at: https://www.mdpi.com/article/10.3390/bios13110983/s1, Figure S1: XPS spectra for C1s; Figure S2: XPS spectra for O1s; Figure S3: XPS spectra for Au4d_3/2_; Figure S4: XPS spectra for Au_4f_; Figure S5: XPS spectra for S2p; Figure S6: XPS spectra for P2p_1/2_; Figure S7: (A) The SPR sensor chip (-COOH) activation with EDC/NHS and (B) SPR signal for -COOH sensor functionalization with 5-HT before aptamer binding; Figure S8: Real-time LSPR sensorgram; Figure S9: Bode plot to determine optimal frequency for Single Frequency EIS operation; Figure S10: Microfluidic setup for dynamic electrochemical sensing; Figure S11: Comparison between N_2_ purged buffer and buffer without N_2_ purging on a bare gold electrode.
