# Supplementary material for: Continuous Real-Time Detection of Serotonin Using an Aptamer-Based Electrochemical Biosensor
Source: Biosensors (Basel). 2023 Nov 13;13(11):983. doi: 10.3390/bios13110983 (PMC10669129; doi:10.3390/bios13110983)
Supplement: Supplementary file 1 [file biosensors-13-00983-s001.zip › biosensors-2677695-supplementary.pdf]

Supplementary Information

Continuous Real-Time Detection of Serotonin Using Aptamer-Based Electrochemical Biosensor

Habib M. N. Ahmad [1], Arturo Andrade [2,3] and Edward Song [1,*]

1 Department of Electrical & Computer Engineering, University of New Hampshire, Durham, NH 03824, USA; habib.ahmad@unh.edu

2 Robert J. & Nancy D. Carney Institute for Brain Science, Brown University, Providence, RI 02912, USA; arturo_andrade@brown.edu

3 Department of Neuroscience, Brown University, Providence, RI 02912, USA

* Correspondence: edward.song@unh.edu

Section S1

The position and intensity of the peaks on the scans are used in XPS analysis. The elemental and chemical composition is shown by the location of the peak on the x-axis, and the height of the peak on the y-axis represents how much of a specific element is evident at the surface. In this section, six most important spectra i.e; C1s, O1s, Au4d$_{3/2}$, Au4f, S2p, P2p$_{1/2}$ associated with proposed sensor surface analysis are presented.

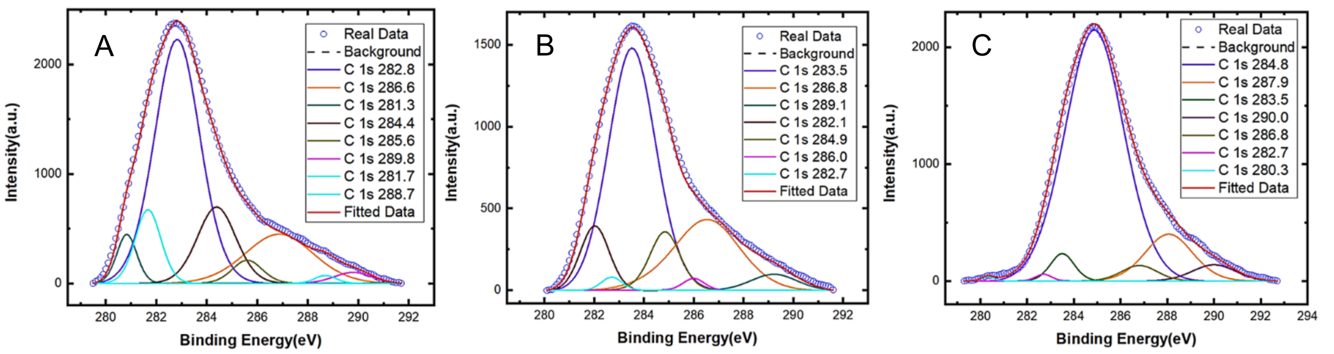

Figure S1: XPS spectra for bare gold electrode (A) and aptamer attached gold electrode before (B) and after (C) exposed to 5-HT for C1s. Each figure contains real data (blue hollow circle), fitted data (red curve), and background (black dashed curve).

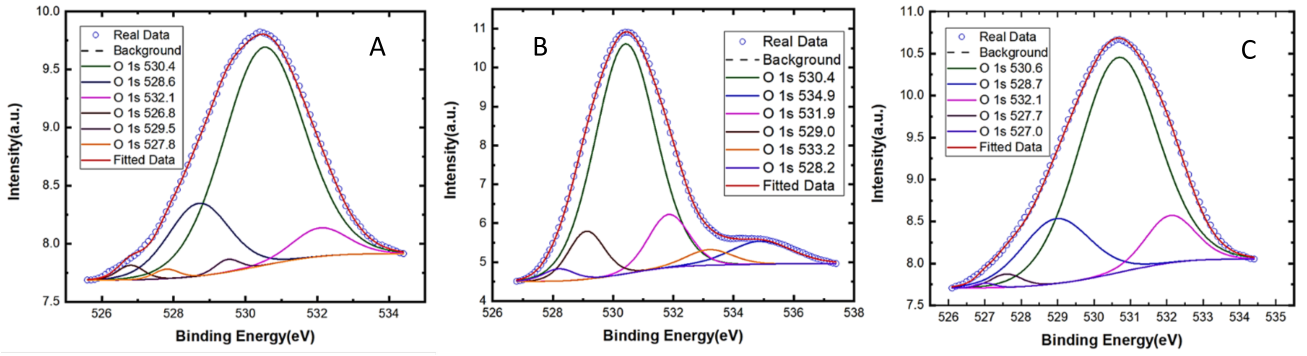

Figure S2: XPS spectra for bare gold electrode (A) and aptamer attached gold electrode before (B) and after (C) exposed to 5-HT for O1s. Each figure contains real data (blue hollow circle), fitted data (red curve), and background (black dashed curve).

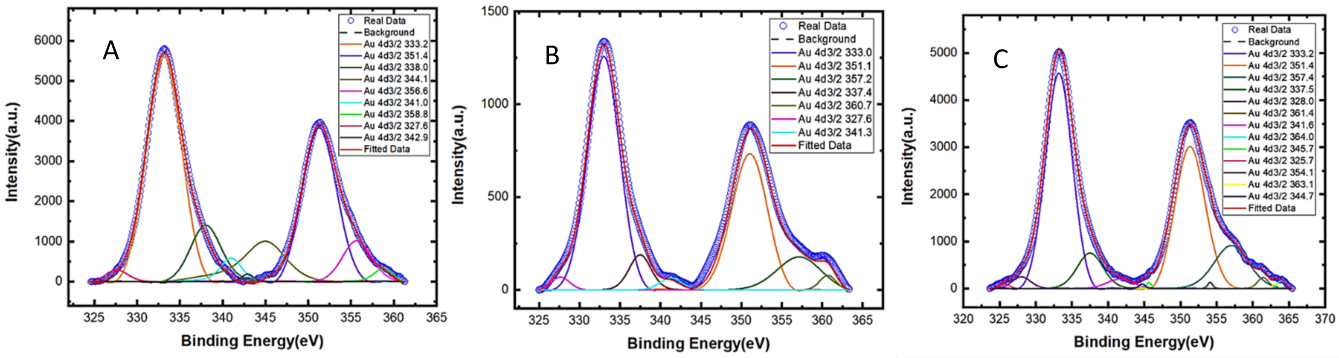

Figure S3: XPS spectra for bare gold electrode (A) and aptamer attached gold electrode before (B) and after (C) exposed to 5-HT for Au4d$_{3/2}$. Each figure contains real data (blue hollow circle), fitted data (red curve), and background (black dashed curve).

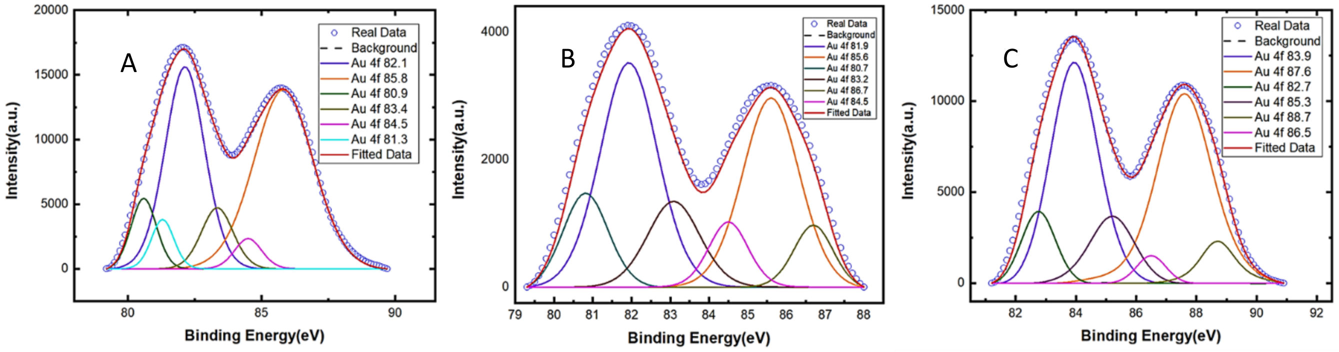

Figure S4: XPS spectra for bare gold electrode (A) and aptamer attached gold electrode before (B) and after (C) exposed to 5-HT for Au4f. Each figure contains real data (blue hollow circle), fitted data (red curve), and background (black dashed curve).

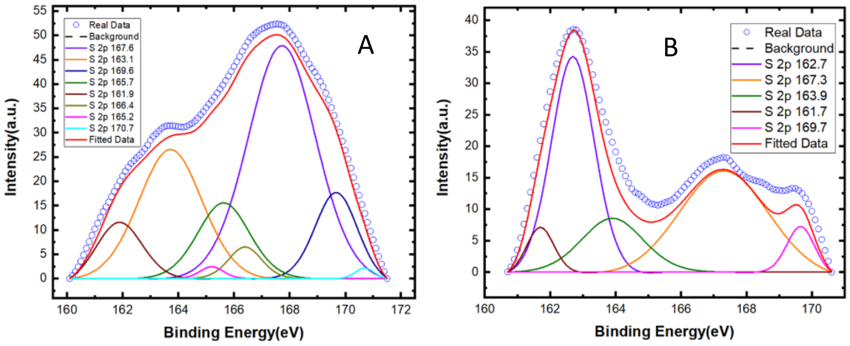

Figure S5: XPS spectra for aptamer attached gold electrode before (A) and after (B) exposed to 5-HT for S2p. Each figure contains real data (blue hollow circle), fitted data (red curve), and background (black dashed curve).

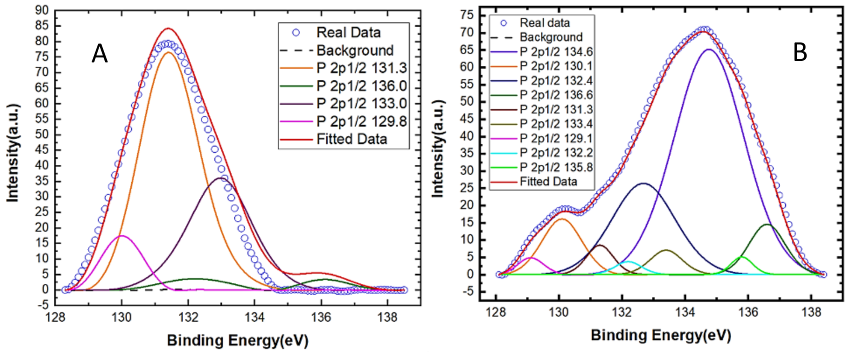

Figure S6: XPS spectra for aptamer attached gold electrode before (A) and after (B) exposed to 5-HT for $P2p_{1/2}$. Each figure contains real data (blue hollow circle), fitted data (red curve), and background (black dashed curve).

Section S2

Surface plasmon resonance (SPR) technique is extremely beneficial for real-time monitoring of biomolecular binding interactions. Its benefits over other approaches encompass real-time monitoring, label-free detection, a modest size of the sample needed, disposable sensor chips, the utilization of complicated samples, and shorter experimental sessions. This section shows SPR chip functionalization and sensorogram for 5-HT-aptamer conjugate.

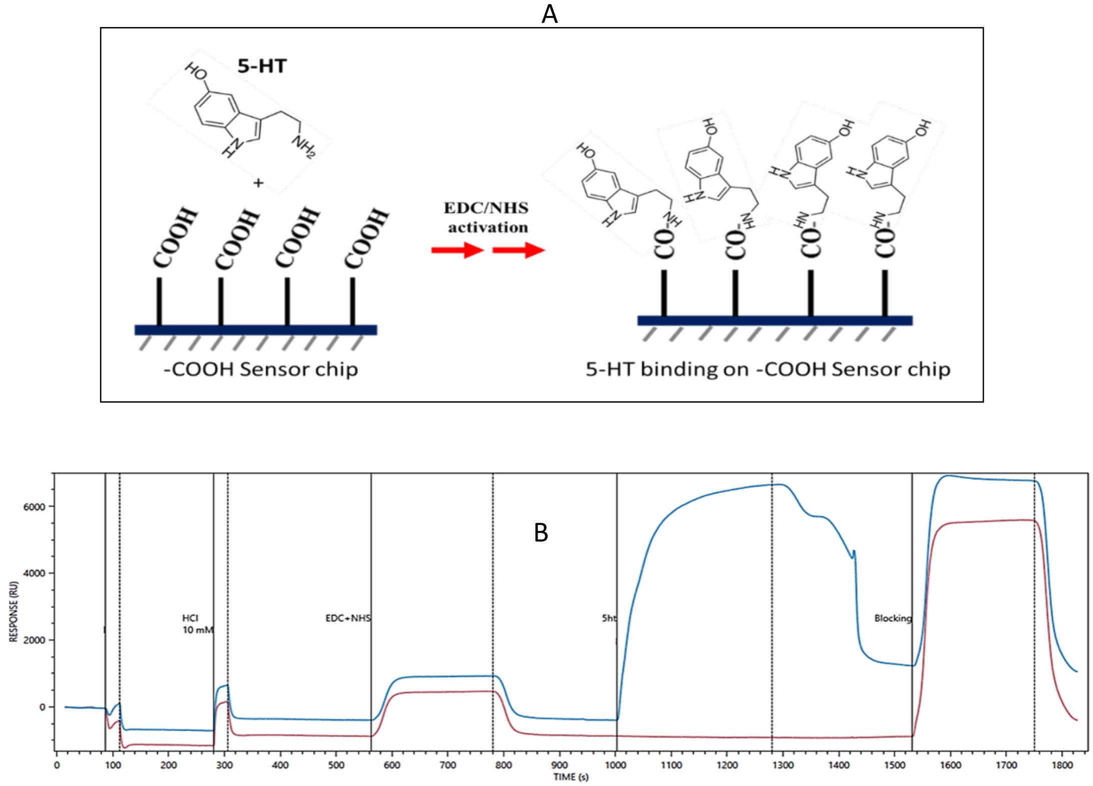

Figure S7: (A) Chemistry behind -COOH chip activation with EDC/NHS; (B) SPR signal for -COOH sensor functionalization with 5-HT before aptamer binding

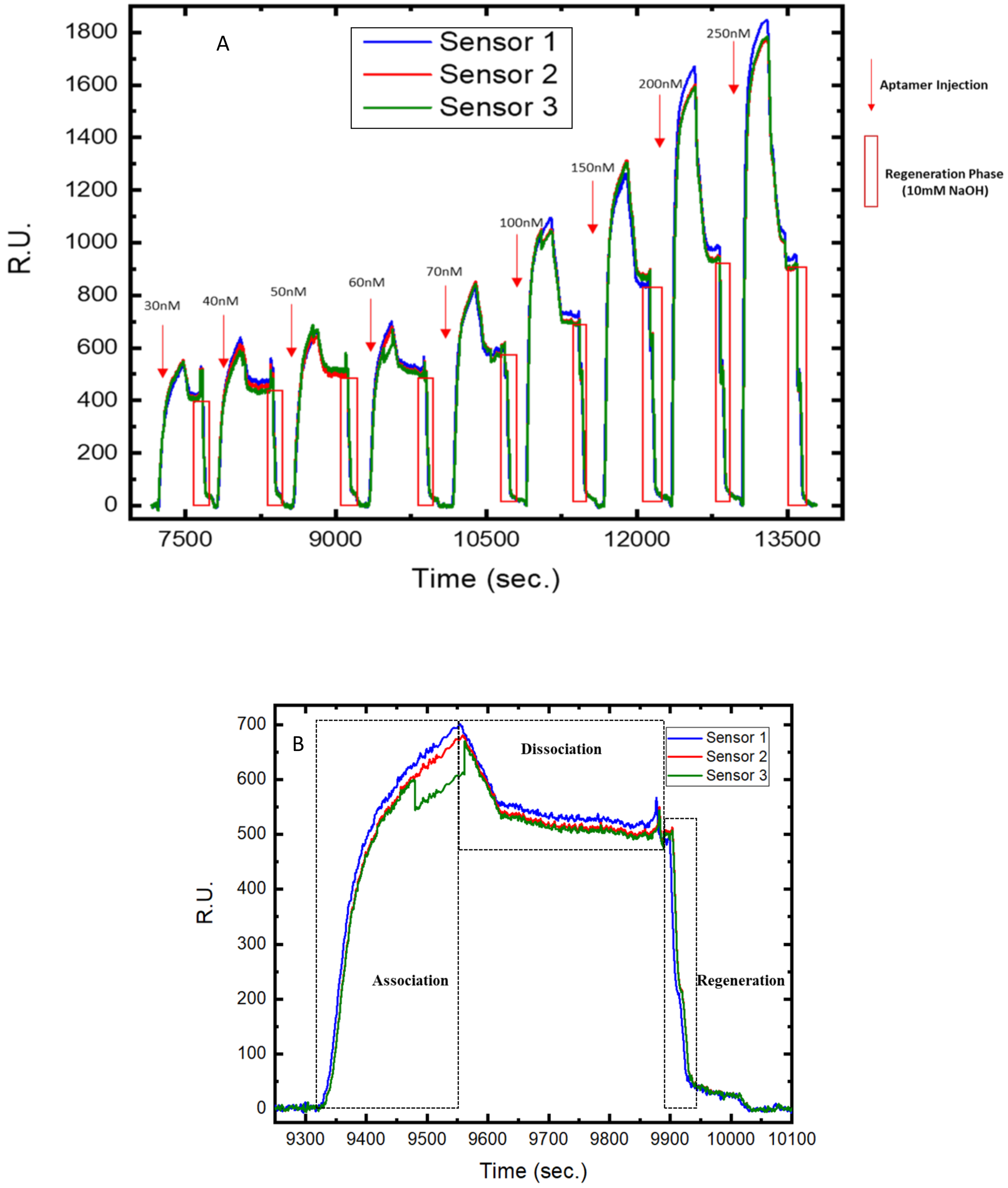

Figure S8: Real-time LSPR Sensorgram: (A) Concentration dependent 5-HT-aptamer conjugate response vs time; (B) Extended view of a portion of Sensorgram (60nM aptamer solution injection) which shows association, dissociation, and regeneration phase .

Section S3

For continuous monitoring of 5-HT concentration, a microfluidic platform was used, and a Single Frequency EIS technique was applied. The Bode plots below shows how the single frequency was selected from the static sensing of 5-HT results.

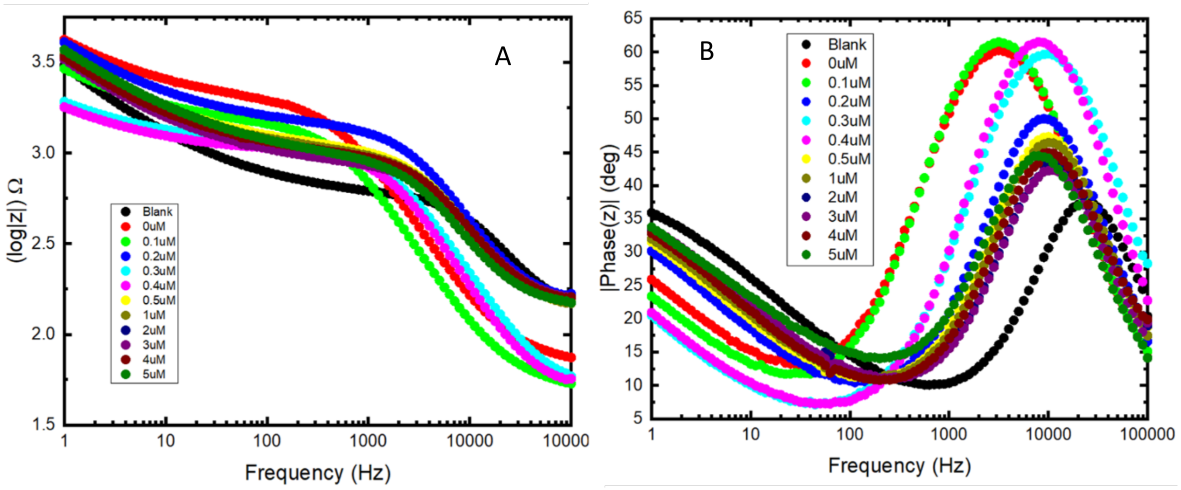

Figure S9: Bode plot to determine frequency point for Single Frequency EIS operation for microfluidic setup: (A) Magnitude vs Frequency; (B) Phase vs Frequency.

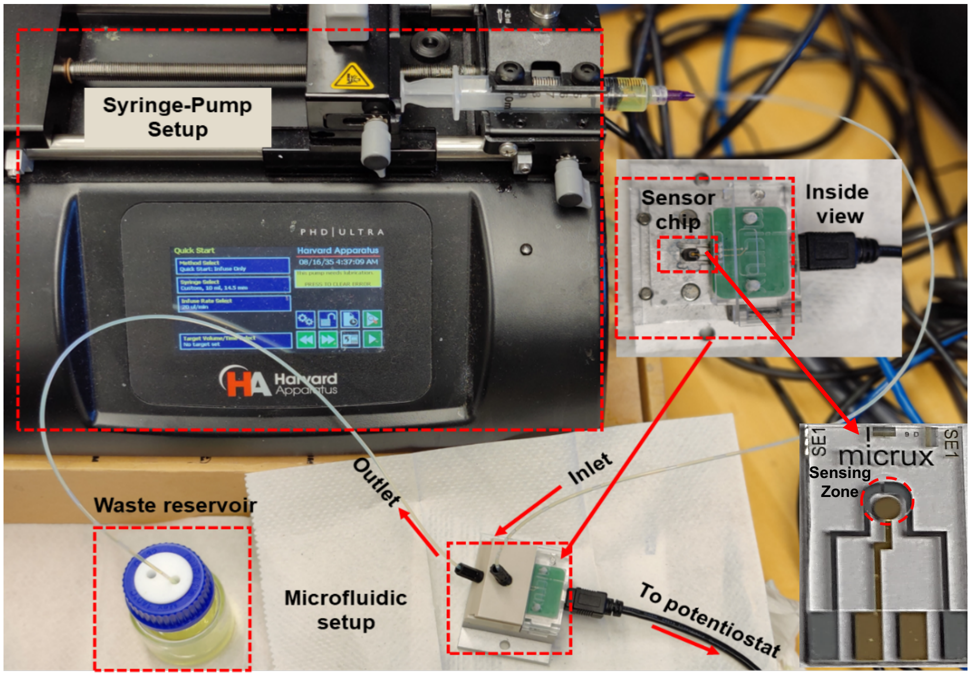

Figure S10: Microfluidic setup for dynamic electrochemical sensing.

Section S4

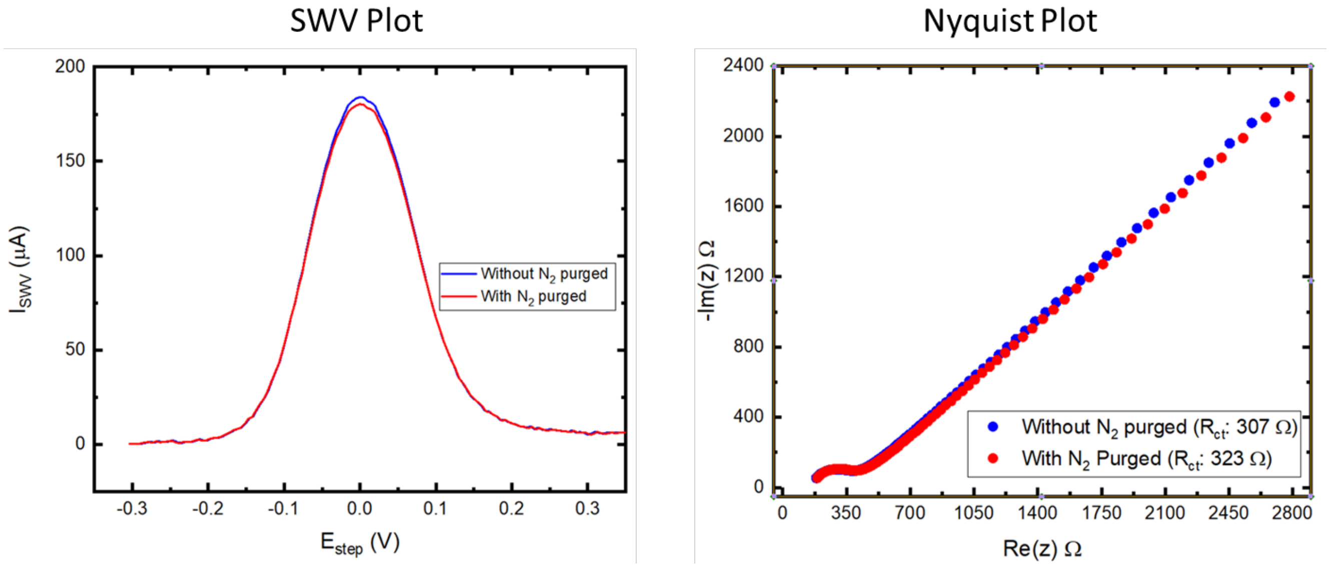

Figure S11: Comparison between N_2 purged buffer (red) and buffer without N_2 purging (blue) on a bare gold electrode.
